# Prevalence and predictors of timely initiation of breastfeeding in Ghana: an analysis of 2017–2018 multiple indicator cluster survey

**DOI:** 10.1186/s13006-021-00383-3

**Published:** 2021-04-17

**Authors:** Paschal Awingura Apanga, Maxwell Tii Kumbeni

**Affiliations:** 1 University of Nevada, Reno, School of Community Health Sciences, Reno, USA; 2 Ghana Health Service, Nabdam District Health Directorate, Nangodi, Ghana

**Keywords:** Timely initiation, Breastfeeding, Predictors, Mothers, Prevalence, Ghana

## Abstract

**Background:**

Timely initiation of breastfeeding is putting the newborn baby to the breast within 1 h of birth. Its practice can prevent neonatal and under-5 mortality. This study aims to assess the prevalence and factors associated with timely initiation of breastfeeding among mothers in Ghana.

**Methods:**

We used data from the 2017–2018 Ghana multiple indicator cluster survey and our analysis was restricted to 3466 mothers who had a live birth within 2 years. Multivariable logistic regression was used to estimate the factors associated with timely initiation of breastfeeding whilst adjusting for potential confounders, and accounted for clustering, stratification, and sample weights.

**Results:**

The prevalence of timely initiation of breastfeeding was 52.3% (95% CI 49.7%, 54.9%). Mothers who were assisted by a skilled attendant at birth had 65% higher odds of timely initiation of breastfeeding compared to mothers who were not assisted by a skilled attendant (adjusted prevalence odds ratio [aPOR] 1.65; 95% CI 1.28, 2.13). Mothers who delivered by Caesarean section had 74% lower odds of timely initiation of breastfeeding compared to mothers who had vaginal delivery (aPOR 0.26; 95% CI 0.18, 0.36). Mothers who had planned their pregnancy had 31% higher odds of timely initiation of breastfeeding compared to mothers who had an unplanned pregnancy (aPOR 1.31; 95% CI 1.05, 1.63). There were also 74% and 51% higher odds of timely initiation of breastfeeding among mothers who perceived their baby was large (aPOR 1.74; 95% CI 1.34, 2.26), and of average size (aPOR 1.51, 95% CI 1.16, 1.97) at birth respectively, compared to mothers who perceived their baby was small.

**Conclusions:**

Interventions to increase timely initiation of breastfeeding should provide breastfeeding support to mothers who have had a Caesarean section, small sized babies and unplanned pregnancies, and to promote birthing by skilled birth attendants.

## Background

Appropriate child feeding practice involves timely initiation of breastfeeding, 6 months of exclusive breastfeeding and complementary feeding from 6 to 24 months of the child’s age, even though breastfeeding may continue to more than 2 years of age [1]. Timely initiation of breastfeeding is putting the newborn to the breast within 1 h of birth [2]. Colostrum is the first breast milk and its ingestion by the newborn within the first hour of life confers passive and active immunity to the neonate against a wide variety of pathogens [3]. The rich protective factors in colostrum can reduce neonatal mortality when given to the newborn within the first hour of birth [4]. The risk of neonatal mortality is high when breastfeeding is not initiated within the first hour [5]. Colostrum also helps develop a healthy gut microbiome which can have long term health benefits [6]. Timely initiation of breastfeeding also stimulates the release of oxytocin and enables contraction of the uterus and decreases postpartum haemorrhage [7]. It also facilitates mother-infant bonding and is positively associated with exclusive breastfeeding practice [8].

In spite of the benefits of timely initiation of breastfeeding, globally only 39% of mothers initiated breastfeeding within the first hour of life, and this varies across regions [9]. Studies have found that factors such as household wealth, maternal education and place of residence are positively associated with the prevalence of timely initiation of breastfeeding [4, 8, 10–13], but these findings have been inconsistent. Whilst John et al. found that wealthier mothers were more likely to initiate breastfeeding within the first hour of birth than their poorer counterparts, Khanal and his colleagues observed that mothers from poor household were more likely to timely initiate breastfeeding [11, 13]. Studies in Zimbabwe and Bangladesh also found that mothers from rural settings timely initiated breastfeeding compared to their peers from urban households [10, 12], but these were contrary to findings in Ethiopia and Nigeria [14, 15].

In Ghana, the policy on breastfeeding advocates for mothers to timely initiate breastfeeding, practice 6 months of exclusive breastfeeding followed by introduction of complementary foods with continued breastfeeding until the child is 2 years old and beyond [16]. Edmond et al. in Ghana observed that 22% of neonatal deaths can be saved if mothers initiate breastfeeding within the first 1 h of birth [4]. Whilst timely initiation of breastfeeding plays an important role in reducing neonatal mortality in Ghana, studies on factors associated with timely initiation of breastfeeding in Ghana are limited [17]. There is also considerable variation in the prevalence of timely initiation of breastfeeding in Ghana [17–20], and most of these studies were not nationally representative [18–20]. Therefore, our primary aim was to assess the prevalence and predictors of timely initiation of breastfeeding among mothers using the most recent multiple indicator cluster survey (MICS). The findings of this study will be relevant in designing appropriate interventions to improve timely initiation of breastfeeding in Ghana.

## Methods

### Study design, study population and data collection

We used data from the MICS conducted in Ghana from 2017 to 2018. The MICS is a representative household survey, which provides national data on women and children with assistance from the United Nations Children’s Fund [21]. The study population was made up of women of reproductive age (15–49 years) with a live birth within 2 years. The MICS uses a two-stage sampling procedure which involves selection of census enumeration areas from each sampling strata using a probability proportionate to the number of households in an enumeration area. The second stage of sampling employs a systematic random sampling to select households from each enumeration area to form survey clusters. Detailed description of the MICS sampling design and procedures are published elsewhere [22, 23].

### Primary outcome

Our primary outcome of interest was timely initiation of breastfeeding. Timely initiation of breastfeeding was defined as a mother who puts her baby to the breast within 1 h of birth [21].

### Predictors

The predictors in our study were antenatal care attendance, delivery assisted by a skilled attendant, mode of delivery, planned pregnancy and perceived delivery size of baby. We compared mothers who had received at least one antenatal care to mothers who did not receive antenatal care. We also compared mothers who perceived the size of their baby at birth was larger than average, and mothers who perceived their baby was of average size to mothers who perceived their babies were smaller than the average size. Other predictors were categorized as: delivery assisted by a skilled attendant (yes, no); mode of delivery (Caesarean section, vaginal delivery); and planned pregnancy (yes, no). A skilled attendant was any health professional such as a doctor, midwife or nurse who assisted the mother at delivery.

### Covariates

Our covariates of interest were: mother’s age, marital status, educational level of mothers, household wealth and place of residence. Mother’s age was categorized as 15–24, 25–34 and 35–49 years. Marital status was categorized as married/cohabitation and never married. The educational level of mothers was categorized as no formal education, primary, secondary and college or higher education. We used household wealth quintiles to construct upper two, middle and lower two wealth quintiles which were used to denote high income, middle income, and poor income households respectively. Place of residence was either rural or urban.

The variable selection for our study was based on previous studies [8, 14, 15, 24], and data available in MICS.

### Data analysis

We conducted descriptive statistics and logistic regression analysis using SAS version 9.3 (SAS Institute, Cary, NC). Descriptive statistics were used to assess the prevalence and characteristics of study population. Complex survey univariate logistic and multivariable logistic regression models were used to assess the relationship between predictor variables and our primary outcome of interest. In the multivariable logistic regression analysis, we adjusted for potential confounders (i.e. covariates) as a priori, as we believe there is a biological plausibility that they might be associated with both predictor variables and primary outcome. We accounted for clustering, stratification, and applied sampling weights to ensure representativeness of our results and also tested for multicollinearity of our adjusted model using pairwise correlation matrix, variance inflation factor and tolerance to ensure that there was no issue of multicollinearity. A *P* - value of less than 0.05 was considered statistically significant.

## Results

### Characteristics of the study population and prevalence of timely initiation of breastfeeding

**Fig. 1:**
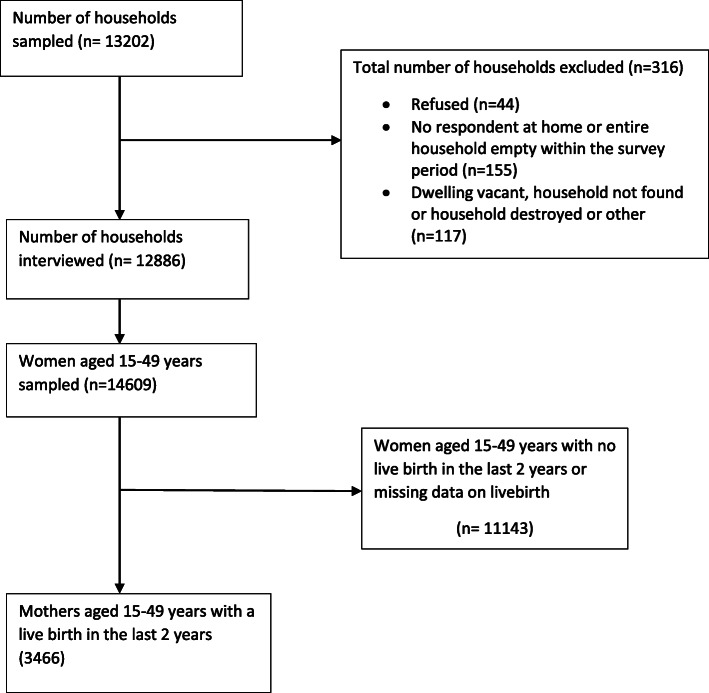
Flowchart showing selection of analytic sample of mothers with a live birth in the past two years that were included in the analysis from Ghana MICS 2017–2018

The study population was made up of 3466 mothers with a live birth in the past 2 years (Fig. 1). The number of hospitals in our study that served as a place of delivery for mothers were made up of 1487 Government hospitals and 209 private hospitals. The mean age of mothers was 28.6 years (results not shown). Many of the mothers in our study were aged 25–34 years, 1516 (47.2%), and 2847 (83.0%) were married/cohabitation. Majority of the mothers had a secondary level of education, 1708 (51.2%). Most of the mothers were from poor income households, 1741 (41.6%), and more than half of them were living in rural settings, 2143 (57.7%) (Table 1).

**Table 1:** Characteristics of the study population (*n* = 3466)

Characteristics	Frequency (%)	^**a**^ Prevalence, ***n*** (%; 95% CI)
**Total sample**	3466 (100)	1857 (52.3; 49.7, 54.9)
**Age (years)**		
15–24	1158 (27.5)	603 (50.7; 46.5, 55.0)
25–34	1516 (47.2)	821 (53.5; 50.1, 56.8)
35–49	792 (25.4)	433 (51.7; 46.8, 56.6)
**Marital status**		
Never married	619 (17.0)	301 (48.1; 42.7, 53.5)
Married/cohabitation	2847 (83.0)	1556 (53.1; 50.3, 56.0)
**Education**		
No formal education	928 (22.1)	510 (52.0; 47.6, 56.4)
Primary	666 (21.3)	341 (50.3; 44.5, 56.1)
Secondary	1708 (51.2)	924 (53.5; 50.0, 57.1)
College or higher education	164 (5.4)	82 (48.9; 36.4, 61.4)
**Household wealth**		
Poor	1741 (41.6)	952 (52.6; 49.3, 55.9)
Middle income	634 (19.5)	325 (52.4; 47.0, 57.7)
High income	1091 (38.9)	580 (51.8; 47.0, 56.6)
**Place of residence**		
Urban	1323 (42.3)	691 (50.3; 46.0, 54.7)
Rural	2143 (57.7)	1166 (53.7; 50.5, 56.8)
**Attended Antenatal care**		
0	95 (2.6)	42 (35.3; 22.7, 48.0)
≥ 1	3371 (97.4)	1815 (52.7; 50.0, 55.4)
**Delivery assisted by skilled attendant**		
No	784 (21.7)	361 (45.8; 41.2, 50.3)
Yes	2681 (78.3)	1495 (54.0; 50.9, 57.2)
**Mode of delivery**		
Vaginal delivery	3046 (87.1)	1735 (55.8; 53.2, 58.4)
Caesarean section	420 (12.9)	122 (27.8; 21.6, 33.9)
**Planned pregnancy**		
No	1701 (50.6)	874 (49.0; 45.1, 52.9)
Yes	1765 (49.4)	983 (55.6; 52.3, 58.9)
**Perceived delivery size of baby**		
Smaller than Average	581 (16.8)	267 (40.8; 35.1, 46.6)
Average	1254 (34.6)	677 (52.7; 48.8, 56.7)
Larger than average	1607 (48.6)	900 (55.7; 52.2, 59.3)

^a^ = Prevalence of timely initiation of breastfeeding; *n* = frequency

The overall prevalence of timely initiation of breastfeeding was 52.3% (95% CI 49.7%, 54.9%) with 1.30% (95% CI 0.79%, 1.81%) of mothers not breastfeeding at all. The prevalence of timely initiation of breastfeeding was similar among mothers that were never married, 48.1% (95% CI 42.7%, 53.5%) and mothers that were married/cohabitation, 53.1% (95% CI 50.3%, 56.0%). This was also similar among mothers with different levels of education: no formal education (52.0%; 95% CI 47.6%, 56.4%); primary (50.3%; 95%: 44.5%, 56.1%); secondary (53.5%; 95% CI 50.0%, 57.1%); and college or higher education (48.9%; 95% CI 36.4%, 61.4%). The prevalence of timely initiation of breastfeeding was also comparatively similar among mothers living in rural settings, 53.7% (95% CI 50.5%, 56.8%) to those in urban settings, 50.3% (46.0%, 54.7%). We did however observe that the prevalence of timely initiation of breastfeeding was higher among mothers that had a planned pregnancy (55.6%; 95% CI 52.3%, 58.9%), received antenatal care (52.7%; 95%, CI 50.0%, 55.4%), had a vaginal delivery (55.8%; 95% CI 53.2%, 58.4%), and were assisted by a skilled attendant (54.0%; 95% CI 50.9%, 57.2%), compared to mothers who did not plan their pregnancy (49.0%; 95% CI 45.1%, 52.9%), received no antenatal care (35.3%; 95% CI 22.7%, 48.0%), had a caesarean section (27.8%; 95% CI 21.6%, 33.9%), and were not assisted by a skilled attendant (45.8%; 95% CI 41.2%, 50.3%), respectively (Table 1).

### Predictors of timely initiation of breastfeeding

The multivariable logistic regression results showed that mothers who were assisted by a skilled attendant during delivery had 65% higher odds of timely initiation of breastfeeding as compared to mothers who were not assisted by a skilled attendant at birth (adjusted prevalence odds ratio [aPOR] 1.65; 95% CI 1.28, 2.13). Mothers who had a Caesarean section had 74% lower odds of timely initiation of breastfeeding compared to mothers who had a vaginal delivery (aPOR 0.26; 95, 95% CI 0.18, 0.36). Mothers whose pregnancies were planned had 31% higher odds of timely initiation of breastfeeding compared to their peers who had unplanned pregnancies (aPOR 1.31; 95% CI 1.05, 1.63). Mothers who perceived that their babies were larger than average size (aPOR 1.74; 95% CI 1.34, 2.26), and of average size (aPOR 1.51; 95% CI 1.16, 1.97), at birth had 74% and 51% higher odds of timely initiation of breastfeeding respectively, compared to mothers who perceived their babies were smaller than average size at birth. However, the age of a mother, educational level, household wealth, marital status, antenatal care attendance and place of residence were not associated with timely initiation of breastfeeding (Table 2).

**Table 2:** Factors associated with timely initiation of breastfeeding (*n* = 3466)

Variable	Unadjusted OR (95% CI)	Adjusted OR (95% CI)
**Age (years)**		
15–24	1	1
25–34	1.12 (0.91, 1.37)	1.10 (0.87, 1.40)
35–49	1.04 (0.81, 1.33)	1.07 (0.79, 1.44)
**Marital status**		
Never married	1	1
Married/cohabitation	1.22 (0.97, 1.55)	1.16 (0.87, 1.54)
**Education**		
No formal education	1	1
Primary	0.93 (0.7, 1.25)	1.08 (0.79, 1.46)
Secondary	1.06 (0.85, 1.33)	1.28 (0.96, 1.71)
College or higher education	0.88 (0.52, 1.50)	1.24 (0.76, 2.04)
**Household wealth**		
Poor	1	1
Middle income	0.99 (0.76, 1.29)	0.96 (0.73, 1.28)
High income	0.97 (0.77, 1.23)	0.98 (0.72, 1.34)
**Place of residence**		
Urban	1	1
Rural	1.14 (0.92, 1.42)	1.18 (0.92, 1.53)
**Attended antenatal care**		
0	1	1
≥ 1	2.04 (1.18, 3.52)	1.63 (0.93, 2.84)
**Delivery assisted by skilled attendant**		
No	1	1
Yes	1.39 (1.11, 1.75)	1.65 (1.28, 2.13)*
**Mode of delivery**		
Vaginal delivery	1	1
Caesarean section	0.31 (0.22, 0.42)	0.26 (0.18, 0.36)*
**Planned pregnancy**		
No	1	1
Yes	1.3 (1.07, 1.59)	1.31 (1.05, 1.63)*
**Perceived delivery size of baby**		
Smaller than average	1	1
Average	1.62 (1.24, 2.11)	1.51 (1.16, 1.97)*
Larger than average	1.82 (1.41, 2.36)	1.74 (1.34, 2.26)*

* = Significant at *P* - value < 0.05; 1 = Reference category

## Discussion

Our study found that overall, 52.3% of mothers had initiated breastfeeding within the first hour of birth. Our results showed that planned pregnancy, delivery assisted by a skilled attendant and perceived size (average\large) of a baby were positively associated with timely initiation of breastfeeding, whilst Caesarean section was negatively associated with timely initiation of breastfeeding. However, age, marital status, education, household wealth, antenatal care attendance and place of residence were not associated with timely initiation of breastfeeding.

The overall prevalence of timely initiation of breastfeeding in our study is higher than the reported overall prevalence of timely initiation of breastfeeding in the West African and Central African sub regions [25]. Timely initiation of breastfeeding in Ghana has increased from 45.9% in 2011 to 52.3% in 2018 [26]. Despite Ghana’s modest improvement in the prevalence of timely initiation of breastfeeding over the 7 year period, the prevalence is still markedly lower than its target of ensuring that 85% of all babies are breastfed within the first 1 h of birth [27]. It has been predicted by Duodu et al. that Ghana may meet its target of timely initiation of breastfeeding by 2044 [17]. Ghana will need to implement breastfeeding programmes whilst prioritizing timely initiation of breastfeeding if the national target is to be achieved on time. The prevalence of timely initiation of breastfeeding in Ghana is also substantially lower than that reported in countries such as Ethiopia (74.3%), Malawi (76.9%), Sudan (69%), Rwanda (81.5%), Liberia (62.3%), Mali (58.7%), Sudan (87.2%), and Mozambique (77.7%) [11, 25, 28–30]. The differences in prevalence of timely initiation of breastfeeding across countries might be attributed to differences in socio-cultural context, economy and health inequalities [11].

We found that mothers whose pregnancies were planned were more likely to initiate breastfeeding within the first hour of birth compared to mothers who had an unplanned pregnancy. This is consistent with a previous study in Turkey [24], and may reflect the poor breastfeeding behavior among mothers who had an unplanned pregnancy [31]. The lower prevalence of timely initiation of breastfeeding among mothers who had an unplanned pregnancy compared to mothers whose pregnancies were planned in our study may be attributed to delayed prenatal care, premature birth and negative physical and mental health effects associated with mothers who do not plan their pregnancies [32–34]. These adverse maternal and child health outcomes might account for the low prevalence of timely initiation of breastfeeding among mothers whose pregnancies were unplanned.

Our findings on the negative association between mothers who had a Caesarean section and timely initiation of breastfeeding is corroborated with many previous studies in low-and middle-income countries [8, 10–12, 17]. There are several possible reasons that might account for the low prevalence of timely initiation of breastfeeding among mothers that had a Caesarean section compared to mothers who had vaginal delivery. Mothers who had a Caesarean section might need some time to recover from anaesthesia and may also have pain adopting to breastfeeding positions [8, 35]. Babies delivered by a Caesarean section might suffer respiratory distress and this can lead to separation from their mothers [8].

Our finding on the positive association between assisted delivery by a skill attendant and timely initiation of breastfeeding was not surprising as we expect health professionals to support mothers to initiate breastfeeding within an hour compared to traditional birth attendants. This finding might also be due to the higher health seeking behaviour among mothers who were assisted by a skilled attendant during delivery compared to mothers who were assisted by traditional birth attendants.

Our analysis showed that antenatal care attendance of at least one visit was not associated with timely initiation of breastfeeding. This finding was inconsistent with results from Mekonen et al. in Ethiopia [14]. The finding in our study might be due to chance, as our observed association between antenatal care attendance and timely initiation of breastfeeding was not significant.

We did also observe in our results that mothers who perceived their babies were of average size or larger than average size at birth were more likely to timely initiate breastfeeding than mothers who perceived their babies were below average size. This finding is in agreement with previous studies [15, 36]. Average or large size babies are often healthy with strong breastfeeding reflexes, and this is associated with timely initiation of breastfeeding [37]. On the contrary small sized babies are often low birthweight, premature and have weak sucking reflexes, poor coordination coupled with difficulty in swallowing [15, 37]. This finding in our study suggests that mothers with small sized infant will require more breastfeeding support to initiate breastfeeding within the first hour of birth. In our results, we also found that marital status, age, education, household wealth, and place of residence were not associated with timely initiation of breastfeeding. Our findings on place of residence, household wealth and education level of the mothers were consistent with results from Bangladesh [38], but were in contrast to many previous studies [8, 11, 28].

The strength of our study is its national representative sample which allows for our findings to be generalized to Ghanaian mothers. However, our study had some limitations. The cross-sectional sample does not allow for our findings to infer causality. Some of our predictor variables were not objectively measured and were subject to measurement error. For example, perceived size of a baby at delivery is not an accurate measure of the weight of a baby. Our predictor variables were also self-reported, and thus are subject to recall bias. We expect recall bias to be similar between mothers who timely initiated breastfeeding, and those who did not. We also expect recall bias on our primary outcome to be similar between exposed and non-exposed predictor variables. We also could not control for potential confounders such as medications that could potentially prevent mothers from timely initiating breastfeeding.

## Conclusions

This study found that planned pregnancy, assisted delivery by a skilled attendant and perceived average or large size of a baby at birth were positively associated with timely initiation of breastfeeding. However, Caesarean section was negatively associated with timely initiation of breastfeeding. Interventions to increase timely initiation of breastfeeding in Ghana should focus on providing breastfeeding support to mothers who have had a Caesarean section and mothers who have had small sized babies. Interventions should also educate women on importance of skilled delivery and to provide breastfeeding support to women with unplanned pregnancies.

## Data Availability

MICS data is publicly available at: https://mics.unicef.org/surveys

## Abbreviations

aPOR: Adjusted prevalence odds ratio
MICS: Multiple indicator cluster survey
